# The Major Autolysin Atl Regulates the Virulence of Staphylococcus aureus by Controlling the Sorting of LukAB

**DOI:** 10.1128/iai.00056-22

**Published:** 2022-03-08

**Authors:** Xuhui Zheng, Sheya Xiao Ma, Amelia St. John, Victor J. Torres

**Affiliations:** a Department of Microbiology, New York University Grossman School of Medicine, New York, New York, USA; b Antimicrobial-Resistant Pathogens Program, New York University Grossman School of Medicine, New York, New York, USA; University of California, Davis

**Keywords:** LukAB, MRSA, *Staphylococcus aureus*, autolysin, cell envelope, cell wall, neutrophils, pore-forming toxins, protein secretion

## Abstract

Infections caused by the Gram-positive bacterium Staphylococcus aureus remain a significant health threat globally. The production of bicomponent pore-forming leukocidins plays an important role in S. aureus pathogenesis. Transcriptionally, these toxins are primarily regulated by the Sae and Agr regulatory systems. However, the posttranslational regulation of these toxins is largely unexplored. In particular, one of the leukocidins, LukAB, has been shown to be both secreted into the extracellular milieu and associated with the bacterial cell envelope. Here, we report that a major cell wall hydrolase, autolysin (Atl), controls the sorting of LukAB from the cell envelope to the extracellular milieu, an effect independent of transcriptional regulation. By influencing the sorting of LukAB, Atl modulates S. aureus cytotoxicity toward primary human neutrophils. Mechanistically, we found that the reduction in peptidoglycan cleavage and increased LukAB secretion in the *atl* mutant can be reversed through the supplementation of exogenous mutanolysin. Altogether, our study revealed that the cell wall hydrolase activity of Atl and the cleavage of peptidoglycan play an important role in controlling the sorting of S. aureus toxins during secretion.

## INTRODUCTION

The Gram-positive bacterium Staphylococcus aureus is commonly found as a colonizer on the skin and mucosae of human host without causing disease (1, 2). Upon gaining access to deeper tissues, S. aureus can cause a wide array of diseases in humans, including skin and soft tissue infections, sepsis, endocarditis, pneumonia, and osteomyelitis (3, 4). The treatment of S. aureus infections is complicated by the emergence of antibiotic resistance. In fact, methicillin-resistant S. aureus (MRSA) is considered a serious threat by the U.S. Centers for Disease Control and Prevention (5).

The current epidemic MRSA lineage in the United States, USA300, is dominant in both community- and hospital-associated MRSA infections (6–9). The versatile lifestyle of USA300 is facilitated by a large repertoire of virulence factors (10). The cytotoxins produced by USA300 play a key role in pathogenesis by forming pores on the targeted host cells, leading to cell death (11). The bicomponent leukocidins are a family of cytotoxins that form hetero-octameric β-barrel pores comprising four pairs of two subunits, designated as slow (S) and fast (F) components based on their chromatography elution profile (12, 13). A single USA300 S. aureus strain can produce up to five leukocidins. These are Panton-Valentine leukocidin (LukSF-PV or PVL), γ-hemolysins HlgAB and HlgCB, leukocidin ED (LukED), and leukocidin AB (LukAB, also known as LukGH) (14). The sequence identity among PVL, HlgAB, HlgCB, and LukED is 60 to 80% within each S-subunit and F-subunit family, while LukAB only shares 30 to 40% sequence identity with the other leukocidins (15). Among these toxins, LukAB has been shown to be the major contributor in killing human phagocytes in tissue-culture infection models with primary human cells (16–20). LukAB targets human cells by recognizing CD11b and HVCN1 as receptors, which dictates its human specificity due to the dissimilarity of these receptors between humans and rodents (21, 22).

While leukocidins are secreted toxins, LukAB is found both secreted and associated with the bacterial cell (17, 18, 23). The sorting of LukAB follows a multistep process controlled by the cell envelope, resulting in differential deposition of the toxin on the bacterial cell or into the extracellular milieu, dependent on growth conditions (23). The cell envelope of S. aureus is composed of the cell membrane, the peptidoglycan cell wall, membrane-anchored lipoteichoic acid (LTA), and cell wall-anchored wall teichoic acid (WTA) (24). The cell membrane is composed of five major types of glycerolipids, phosphatidylglycerol (PG), diacylglycerol (DAG), lysyl-PG (LPG), diglucosyl-DAG (Glc_2_-DAG), and cardiolipin (25, 26). Previously, LPG and Glc_2_-DAG were shown to be important for sorting LukAB from the bacterial cell envelope to the extracellular milieu (23).

The process of cell wall synthesis and turnover in S. aureus is controlled by a series of enzymes. We previously identified autolysin (Atl), the major cell wall hydrolase in S. aureus, as an enzyme involved in the LukAB sorting process (23). Atl is produced as a preproprotein of ∼137 kDa that is composed of a signal peptide for general secretion (Sec)-dependent membrane translocation, a propeptide of unknown function, and two catalytically active domains, amidase (AM) and glucosaminidase (GL) (27). The AM domain is an *N*-acetylmuramyl-l-alanine amidase that cleaves between MurNAc in the peptidoglycan backbone and l-alanine in the stem peptide (28, 29). The GL domain is an exo-β-*N*-acetylglucosaminidase that cuts the glycan backbone, resulting in GlcNAc-MurNAc disaccharide units (30). An S. aureus *atl* mutant is characterized by defects in autolysis activity, daughter cell separation, and attachment to polymer surfaces (31–33).

Here, we characterized the role of Atl in the secretion of LukAB and determined the impact of Atl on the cytotoxicity of S. aureus toward primary human phagocytes. We show that the *atl* mutant exhibits increased cytotoxicity toward primary human polymorphonuclear leukocytes (PMNs), which is the result of increased secretion of LukAB. The regulation of LukAB secretion by Atl is independent of transcription, but it is attributed to the differential sorting of LukAB. We show that the increased secretion of LukAB in the *atl* mutant can be chemically complemented by the addition of mutanolysin, an enzyme that cleaves peptidoglycan and disaggregates the *atl-*deficient cells. These results indicate that the cell wall hydrolase activity of Atl controls the postmembrane sorting of a critical virulence factor in USA300 S. aureus.

## RESULTS

### Increased protein secretion in the *atl* mutant.

**FIG 1:**
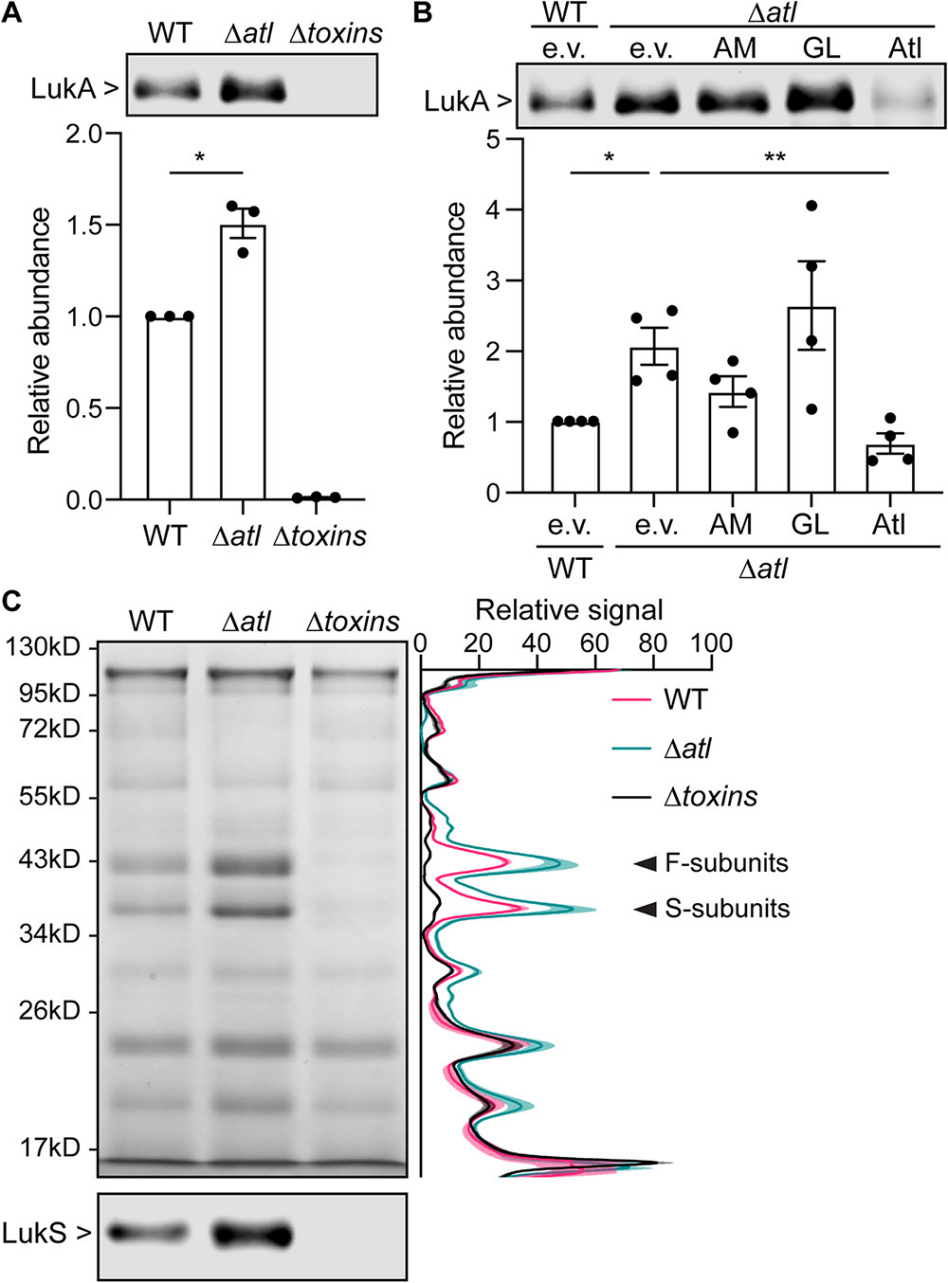
Atl influences the secretion of leukocidins. (A) Immunoblot of LukA in the culture supernatant of WT, the *atl* mutant, and a mutant strain depleted of all pore-forming toxins (*Δtoxins*). A representative immunoblot (top) and the mean ± SEM of the LukA signal from 3 independent experiments are shown (bottom). *, *P ≤ *0.05 comparing WT and the *atl* mutant by a paired *t* test. (B) Immunoblot of LukA in the culture supernatant of WT containing empty vector (e.v.) and the *atl* mutant containing the indicated Atl domains or full-length Atl. A representative immunoblot (top) and the mean ± SEM of LukA signal from 4 independent experiments (bottom) are shown. *, *P ≤ *0.05; **, *P ≤ *0.01 by repeated-measures (RM) one-way analysis of variance (ANOVA) with Tukey’s multiple-comparison tests. (C) Exoprotein profile as detected in an InstantBlue-stained SDS-PAGE (top) and immunoblot of leukocidin S subunits (bottom) of indicated strains. Signals of the InstantBlue-stained SDS-PAGE from 3 independent experiments were quantified and plotted on the right. Shaded area indicates SEM. The arrows point to the bands at the size of leukocidins. The immunoblot is a representative image of 3 independent experiments.

Through a genetic screen using a transposon insertion library of USA300 S. aureus, the Nebraska transposon mutant library (34), we noticed that the *atl* transposon mutant produced more LukAB in the culture supernatant than the parental USA300 strain (23). To confirm the results of the screen, we constructed a mutant where the full *atl* open reading frame (ORF) was deleted in USA300, and we examined the secretion of LukAB in this mutant by immunoblotting. Indeed, the *atl* mutant showed ∼50% increase in the level of LukA, a proxy for LukAB, in the culture supernatant compared to wild type (WT) (Fig. 1A). A mutant strain lacking all leukocidins and *hla* (Δ*toxins*) was included as a negative control (Fig. 1A). We took advantage of a previously reported plasmid expression system for complementation studies (33). The production of full-length Atl in the *atl* mutant background restored LukAB secretion to the WT level (Fig. 1B). However, production of the AM or GL domain alone failed to complement the LukAB secretion phenotype (Fig. 1B).

We next examined the role of Atl in protein secretion in USA300. The protein levels in the culture supernatant of early-stationary-phase bacteria were semiquantitatively analyzed by total protein staining. In general, the *atl* mutant secreted a greater amount of exoproteins than WT (Fig. 1C). The most significant difference was observed with the two bands corresponding to the S and F subunits of leukocidins (Fig. 1C, arrows). Because of the high sequence similarity between PVL, LukED, HlgAB, and HlgCB, we used a pan-leukocidin antibody to detect the S subunits of these leukocidins. The immunoblot showed that indeed the leukocidins were oversecreted in the *atl* mutant (Fig. 1C). Taken together, these results demonstrate that Atl negatively regulates the secretion of leukocidins.

### Atl influences the virulence of USA300.

**FIG 2:**
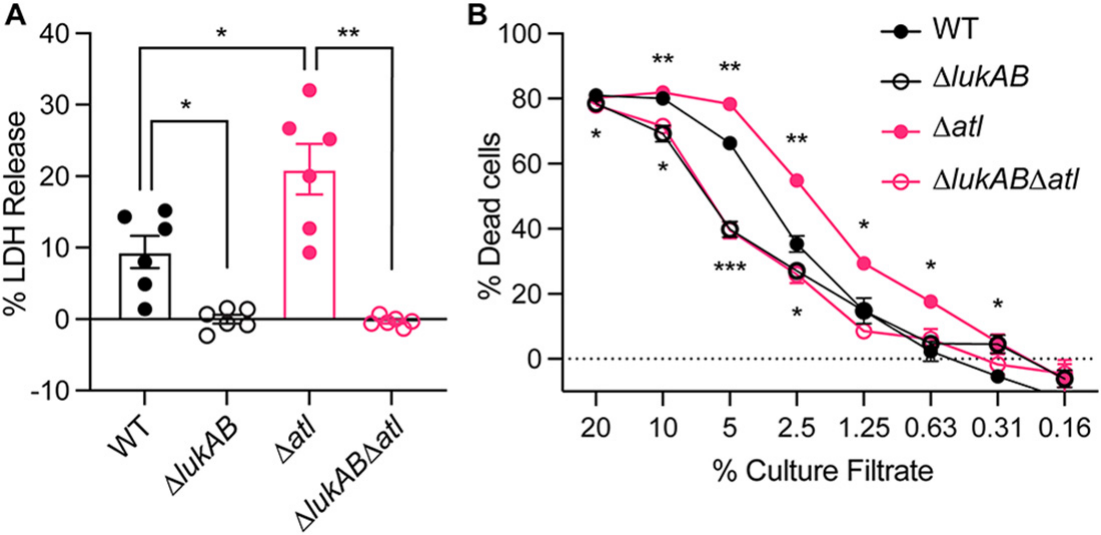
The killing of PMNs by WT and isogenic mutant strains. (A) Infection of PMNs with indicated strains for 2 h with an MOI of 10. PMN lysis was measured by LDH release. Data show mean ± SEM of PMNs isolated from 6 independent blood donors. *, *P ≤ *0.05; **, *P ≤ *0.01 by RM one-way ANOVA with Tukey’s multiple-comparison tests. (B) Intoxication of PMNs with cell-free supernatants from indicated strains. The PMN viability was measured by CellTiter. Data show mean ± SEM of PMNs isolated from 6 independent blood donors. *, *P ≤ *0.05; **, *P ≤ *0.01; ***, *P ≤ *0.001 compared to WT by RM one-way ANOVA with Dunnett’s multiple-comparison tests.

Leukocidins impair host immune responses by directly targeting and killing phagocytes (14, 20, 35, 36). We next sought to examine the virulence potential of the *atl* mutant using a tissue culture model of infection where primary human PMNs were infected with live USA300, and PMN lysis was measured by quantifying the release of the mammalian lactate dehydrogenase (LDH) enzyme. These studies revealed that the *atl* mutant lysed significantly more PMNs than WT USA300 (Fig. 2A). LukAB was responsible for the observed PMN killing by the *atl* mutant, as deletion of *lukAB* in the WT or *atl* mutant resulted in significantly reduced cell death (Fig. 2A).

To examine if the increased LukAB secretion observed in an *atl* mutant contributes to the enhanced cytotoxicity of the *atl* mutant, we incubated primary human PMNs with different concentrations of bacteria-free culture supernatant from USA300 and then evaluated PMN viability by measuring cellular metabolic activity. Consistent with the infection results, the culture supernatant of the *atl* mutant was more cytotoxic than the WT (Fig. 2B). When *lukAB* was deleted from the *atl* mutant, this strain showed the same cytotoxicity as the *lukAB* isogenic mutant of WT (Fig. 2B), indicating that secreted LukAB is responsible for the increased virulence of *atl* in this model.

### Atl does not affect the transcription of leukocidin genes.

**FIG 3:**
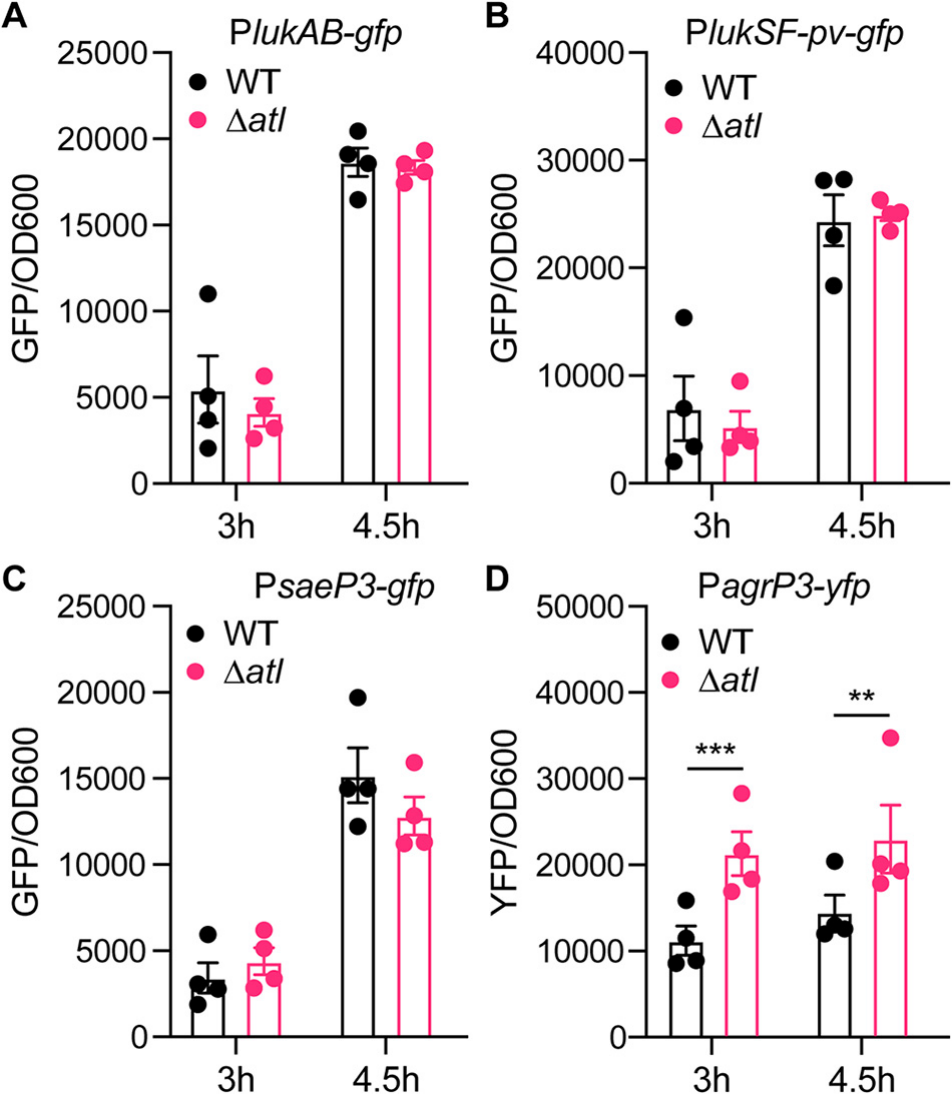
Promoter activities of leukocidins and regulators. Activities of the *lukAB* (A), *lukSF-pv (pvl)* (B), *saeP3* (C), and *agrP3* (D) promoters in the WT and *atl* mutant at the exponential (3 h) and early-stationary (4.5 h) phases. The indicated promoters were fused to *gfp* or *yfp* genes. The promoter activities were measured as fluorescence and normalized by OD_600_. Data show mean ± SEM from 4 independent experiments. *, *P ≤ *0.05; **, *P ≤ *0.01; ***, *P ≤ *0.00 by two-way ANOVA with Sidak multiple-comparison test.

Next, we investigated how the *atl* mutation leads to increased secretion of leukocidins. We first examined whether the *atl* mutation has any transcriptional effect on the leukocidins by measuring the activities of leukocidin promoters in the WT strain and the *atl* isogenic mutant. We chose to evaluate the promoters of *lukAB* and *pvl*, as these two toxins are abundantly produced and their promoter activities are comparable under *in vitro* culture conditions (37, 38). We measured promoter activities at both the exponential (3 h) and early stationary phase (4.5 h), as the transcriptional regulation may occur prior to the protein secretion. At both time points, the promoter activities of *lukAB* and *pvl* remained the same in the *atl* mutant as the WT (Fig. 3A and B).

In addition to the leukocidins, we also observed an increased secretion of other exoproteins in the *atl* mutant (Fig. 1C). To further dissect the effect of Atl on the production of exoproteins, we measured the promoter activities of two master regulators of exoprotein gene expression, *sae* and *agr* (39, 40). The promoter activity of *sae* remained the same in the WT and *atl* mutant (Fig. 3C). However, the promoter activity of *agr* was increased in the *atl* mutant (Fig. 3D), potentially due to the increased local concentration of autoinducing peptides in the unseparated *atl* cell clusters. The increased *agr* activity may lead to greater production of Sae-independent exoproteins such as PSMs (41), which could also promote the release of cytoplasmic proteins (42). In conclusion, the observed increased secretion of leukocidins in the *atl* mutant is not the result of increased transcription of the leukocidin genes.

### Atl controls the sorting of LukAB.

**FIG 4:**
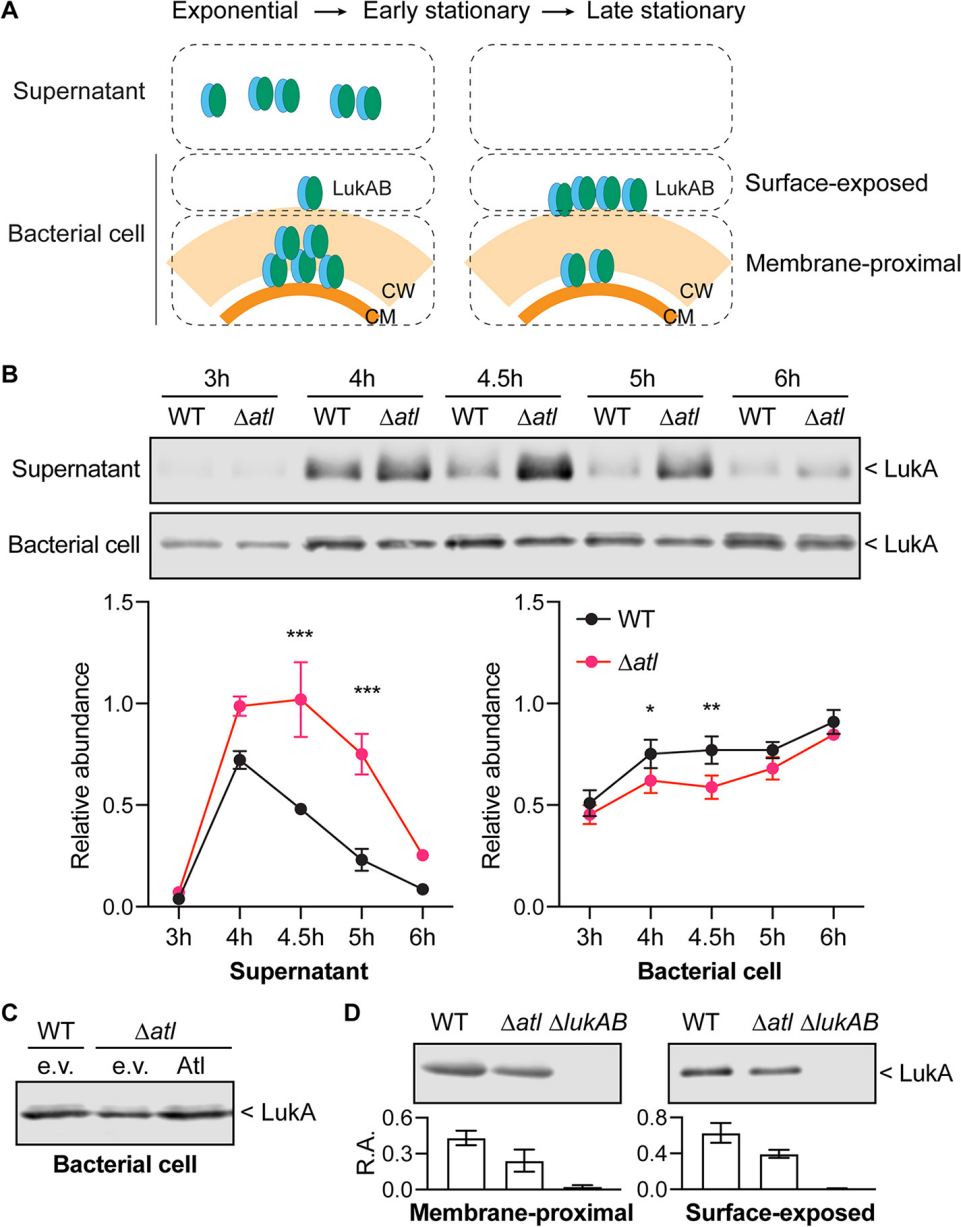
Sorting of LukAB in the *atl* mutant. (A) Diagram of LukAB localization with different compartments of the cell envelope. LukAB is secreted into the culture supernatant at the exponential and early-stationary phases but is absent in the supernatant at the late-stationary phase. LukAB can be found associated with the bacterial cells in all growth phases. The bacterial cell can be further separated into the membrane-proximal and surface-exposed compartments. Most LukAB is found in the membrane-proximal compartment at the exponential phase but in the surface-exposed compartment at the late-stationary phase. CM, cell membrane. CW, cell wall. (B) Representative immunoblots of LukA in the cultures from different growth times (top) and the quantification of the LukA signal in the culture supernatant or associated with the bacterial cell from the immunoblot results (bottom). The signal was normalized to 50 ng purified recombinant LukAB on each membrane. Data show mean ± SEM from 3 independent experiments. *, *P ≤ *0.05; **, *P ≤ *0.01; ***, *P ≤ *0.001 by two-way ANOVA with Sidak multiple-comparison test. (C) Immunoblot of LukA associated with the bacterial cell in WT containing empty vector (e.v.), the *atl* mutant containing empty vector (e.v.), or plasmid-expressed full-length Atl. Figure shows a representative immunoblot from 3 independent experiments. (D) Representative immunoblots (top) and quantifications of the LukA signal (bottom) in the membrane-proximal or surface-exposed compartments associated with the bacterial cell. Relative abundance (R.A.) of LukA signal was normalized to 50 ng purified recombinant LukAB on each membrane. Data show mean ± SEM from 3 independent experiments. *P* values between WT and Δ*atl* determined by two-tailed paired *t* tests as follows: membrane proximal, 0.04; surface exposed, 0.12.

While the other leukocidins are primarily secreted into the extracellular milieu, LukAB is found both secreted and associated with the bacterial cell envelope (17, 18, 23). The secretion of leukocidins is thought to start with translocating across the cell membrane through the Sec pathway (43, 44). After being released from the cell membrane, LukAB can be retained in the cell envelope. The distribution of LukAB in different compartments of the cell envelope and its secretion into the culture supernatant are dependent on the bacterial growth phase (Fig. 4A) and the presence of LPG and Glc_2_-DAG (23). To evaluate if Atl also controls the sorting of LukAB, we examined the presence of LukAB associated with the bacterial cell versus in the culture supernatant at different growth phases (Fig. 4B). Whenever LukAB was detectable in the culture supernatant, more LukAB was found in the *atl* mutant than the WT. On the other hand, the levels of LukAB associated with the bacterial cell were significantly reduced in the *atl* mutant compared to WT (Fig. 4B). The reduction of the bacterial cell-associated LukAB was complemented by producing full-length Atl (Fig. 4C). Thus, Atl negatively regulates LukAB sorting from the cell envelope to the extracellular milieu.

The bacteria-associated LukAB can be further separated into the membrane-proximal and surface-exposed compartments (Fig. 4A). After membrane translocation, LukAB first accumulates in the membrane-proximal compartment before being sorted to the cell surface and extracellular milieu (23). We found that in the *atl* mutant, LukAB levels were reduced in both bacteria-associated compartments compared to WT (Fig. 4D), suggesting that the *atl* mutant is more efficient in releasing LukAB from the cell envelope into the extracellular milieu.

### The LukAB sorting is dependent on the cell wall hydrolase activity.

**FIG 5:**
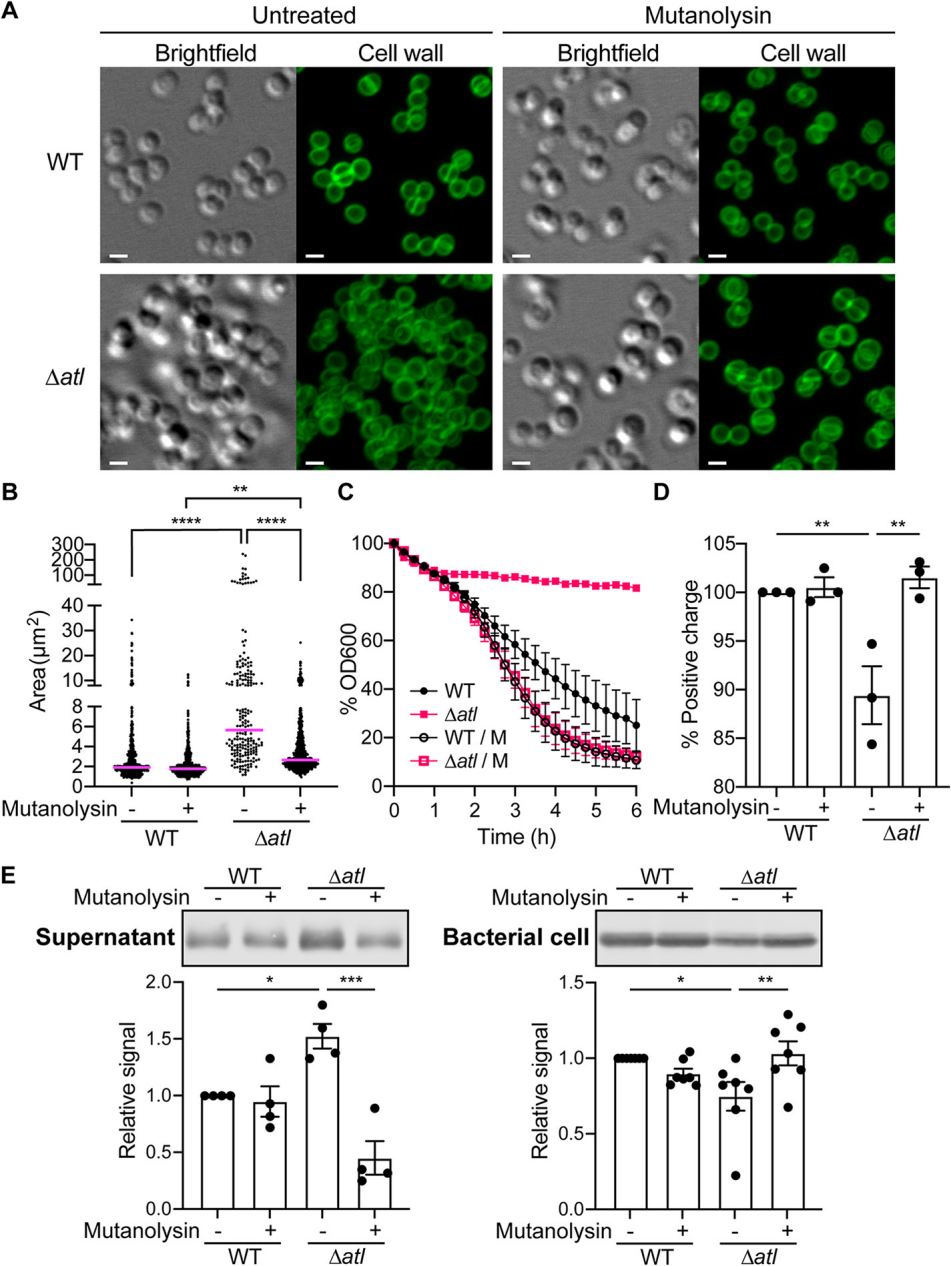
Mutanolysin complements the phenotype of the *atl* mutant. (A) Light microscopy of WT and Δ*atl* cells at the early stationary phase (4.5 h), treated with or without mutanolysin. The cell wall was stained with BODIPY FL vancomycin. Scale bar, 1 μm. (B) Area of the connected cells on a two-dimensional projection of WT and Δ*atl*, treated with or without mutanolysin. Each dot is a cluster of cells. The line indicates the median. **, *P ≤ *0.01; ****, *P < *0.0001 by ordinary one-way ANOVA with Tukey’s multiple-comparison test. (C) Autolysis activity of WT and *Δatl* in 0.05% Triton X-100 in PBS, supplemented with or without mutanolysin. Data show mean ± SEM from 3 independent experiments. (D) Surface positive charge of WT and Δ*atl* measured by cytochrome *c* binding to the cell surface. Data show mean ± SEM from 3 independent experiments. **, *P ≤ *0.01 by RM one-way ANOVA with Tukey’s multiple-comparison test. (E) Representative immunoblots (top) and quantification (bottom) of LukA in the culture supernatant or bacterial cell lysate of WT and Δ*atl* treated with or without mutanolysin. Bars show mean ± SEM from *n* = 4 (supernatant) or *n* = 7 (bacterial cell) independent experiments. *, *P ≤ *0.05; **, *P ≤ *0.01; ***, *P ≤ *0.001 by RM one-way ANOVA with Tukey’s multiple-comparison test.

Atl is a prominent cell wall hydrolase that controls cell wall turnover (30–32). As a result of insufficient cleavage of peptidoglycan, the *atl* mutant cells are connected by peptidoglycan threads, resulting in significant cell clusters (Fig. 5A) (30–32). Mutanolysin, a muramidase produced by Streptomyces globisporus, has been shown to be able to digest S. aureus peptidoglycan threads on the cell surface without causing cell lysis (31). Indeed, by culturing the *atl* mutant with mutanolysin, the cells were efficiently separated (Fig. 5A). Consistent with this, mutanolysin significantly reduced the size of cell clusters in the *atl* mutant, although the median cluster size of the mutanolysin-treated *atl* mutant remained slightly higher than the WT (Fig. 5B).

The autolysis activity of USA300 depends on the presence of cell wall hydrolases. In the *atl* mutant, the Triton X-100-induced autolysis is disrupted (33). However, supplementation with mutanolysin restored the autolysis activity of the *atl* mutant (Fig. 5C). Last, we observed that the changes in peptidoglycan and cell clustering resulted in a reduction in the surface charge of the *atl* mutant, a property that was also reversed by mutanolysin (Fig. 5D). These data established that mutanolysin serves as an exogenous source of cell wall hydrolase that can restore the defects in cell wall turnover exhibited by the *atl* mutant.

To examine if the restoration of cell wall hydrolysis is sufficient to control LukAB sorting in the *atl* mutant, we examined the localization of LukAB in USA300 strains cultured with or without mutanolysin. We observed that when the *atl* mutant was treated with mutanolysin, the secretion and bacterial cell association of LukAB returned to WT levels (Fig. 5E). Thus, the multistep sorting of LukAB depends on proper cleavage of peptidoglycan.

## DISCUSSION

In this study, we examined the contribution of a prominent cell wall hydrolase, Atl, to the sorting of LukAB. We show that in the *atl* mutant, increased amounts of LukAB, as well as other leukocidins, were released into the extracellular milieu. This altered toxin secretion is independent of transcriptional regulation. Instead, our data establish that Atl influences the protein sorting process, as less LukAB was found associated with the cell envelope. Using mutanolysin to complement the phenotype exhibited by the *atl* mutant, our data indicate that the cell wall hydrolase activity of Atl is the key regulator for sorting LukAB from the cell envelope to the extracellular milieu. As a result of enhanced sorting, deletion of *atl* in USA300 results in increased cytotoxicity toward primary human PMNs. Taken together, by studying the role of Atl in sorting LukAB, our study revealed that cell wall hydrolase activity is not only crucial for cell wall turnover but also for the sorting of toxins across the cell wall in S. aureus.

The expression of Atl is tightly regulated by multiple master regulators, such as negative regulators MgrA (45), ArlRS (46), LytSR (47), and SarA (48), as well as the positive regulators Agr (48) and CidAB (49). Posttranscriptionally, Atl is suppressed by the presence of WTA (50, 51). The complex regulatory system of Atl allows S. aureus to respond to different environmental conditions and adjust the rate of cell wall degradation and separation of dividing cells. In addition, the current study demonstrates that the release of virulence factors is also controlled by Atl.

**FIG 6:**
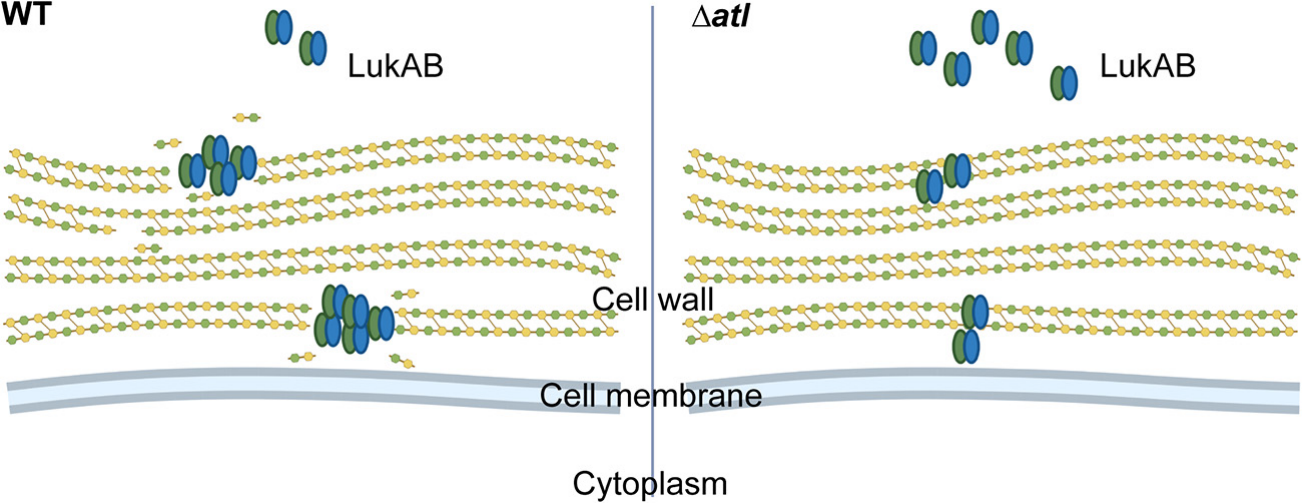
The sorting of LukAB is dependent on Atl cell wall hydrolase activity. Model of how Atl may influence the sorting of LukAB. In WT, the activity of Atl creates depots for LukAB in the cell envelope by breaking down the peptidoglycan strands. In the *atl* mutant, such depots are scarce, and more LukAB is therefore sorted into the extracellular milieu. Created with BioRender.com.

We envision that the activity of Atl creates a depot for LukAB to be associated with the cell envelope (Fig. 6). Spatially, the primary structure of the cell wall of Gram-positive bacteria, peptidoglycan, is a single macromolecule with pores ranging from 6 nm in diameter at the internal surface to 39 nm in diameter at the external surface (52). The elasticity of the cell wall enables bacteria to resist high osmotic pressure, but the plasticity of the cell wall is what allows for cell wall turnover mediated by cell wall synthases and hydrolases in order for bacteria to grow (53). During plastic deformation, depots may be created in the cell envelope for proteins to reside. Meanwhile, active hydrolysis is more likely to generate reactive residues that allow noncovalent interactions such as hydrophobic or electrostatic interactions between LukAB and peptidoglycan fragments. Nevertheless, it is also possible that the Atl activity influences the level of other cell envelope structures that interact with LukAB in the cell envelope.

Previously, Atl has been reported to modulate the expression and secretion of other proteins. In the *atl* mutant, multiple cell wall hydrolases are transcriptionally upregulated and are more abundant in the extracellular milieu, potentially compensating for the loss of *atl* (54). Atl is important for the excretion of a number of cytoplasmic proteins, while the transcription of corresponding genes remains unchanged (54). These proteins lack a signal peptide that allows them to be secreted through canonical pathways. Cell lysis, cell envelope modulation by Atl, and the generation of extracellular vesicles are thought to be the major routes for the release of these cytoplasmic proteins (55). Here, we established the role of Atl in coordinating the secretion of toxins in USA300 and showed that the absence of Atl leads to enhanced secretion of other exoproteins, including leukocidins. Interestingly, we observed an increased *agr* promoter activity but not the promoters of *sae* or leukocidins in the *atl* mutant. The transcription of *lukAB* and *lukSF-PV* is directly regulated by SaeR, while both *sae* and leukocidins are regulated by *agr* through RNAIII and Rot (56–59). The unsynchronized promoter activities may suggest additional regulatory factors involved in toxin expression.

The finding that mutanolysin reverts the phenotypes of the *atl* mutant suggests that the cleavage of peptidoglycan, but not Atl itself, influences the sorting of toxins. A previous study has reported that another cell wall hydrolase, SagB, is also important for the secretion of a subset of proteins, particular adhesins and superantigen-like proteins in S. aureus strain Newman (60). Although Atl contains both AM and GL domains, the AM domain of Atl contributed greater to the murein hydrolase activity (33), which is in agreement with our data that the AM domain showed a partial complementation of LukAB sorting (Fig. 1B). Instead, SagB is the major GL that determines the length of glycan chains in S. aureus (60, 61). It is yet unclear whether SagB influences the sorting of leukocidins in USA300. The accumulation of unprocessed peptidoglycan in the *atl* mutant versus the elongated glycan chains without cross-peptides in the *sagB* mutant may alter distinct aspects of the cell envelope and thus likely differentially contribute to the sorting of selective groups of exoproteins. Studying the contribution of different cell wall hydrolases to the sorting of exoproteins could provide a new avenue to understanding how Gram-positive bacteria transport proteins across the cell wall.

## MATERIALS AND METHODS

### Bacterial growth and culture conditions.

S. aureus strains, plasmids, and primers used in this study are listed in Table 1. S. aureus USA300 strain AH-LAC (62) was used in all experiments as the wild-type (WT) strain. Escherichia coli IM08B (63) was used for cloning and was grown in Luria-Bertani broth with 100 μg/mL ampicillin or 20 μg/mL chloramphenicol when appropriate. S. aureus was routinely grown at 37°C on tryptic soy agar (TSA). For plasmid selection, erythromycin was added at 5 μg/mL, and chloramphenicol was added at 10 μg/mL in the overnight culture or 5 μg/mL in the subculture. When appropriate, mutanolysin was supplemented at 10 U/mL in the subculture.

**TABLE 1:** Bacterial strains, plasmids, and primers used in this study

Strain, plasmid, or primer identifier	Name	Description	Reference or source
Strains			
VJT15.77	AH-LAC (WT)	ErmS USA300 parent strain	62
VJT23.52	Δ*lukAB*	AH-LAC with a clean deletion of the *lukAB* operon	18
VJT58.79	*Δtoxins*	AH-LAC mutant strain lacking all bicomponent leukocidins and α-toxin	71
VJT80.33	*Δatl*	AH-LAC carrying the *tetM* cassette in replacement of the *atl* gene	This study
VJT80.97	*ΔlukAB Δatl*	AH-LAC Δ*lukAB* carrying the *tetM* cassette in replacement of the *atl* gene	This study
Plasmids			
	pOS1-P*lukAB-gfp*	*lukAB* promoter controlling expression of a superfolder GFP	This lab
	pOS1-P*pvl-gfp*	*lukSF-pv* promoter controlling expression of a superfolder GFP	This lab
	pOS1-P*sae-gfp*	*sae* P1 promoter controlling expression of a superfolder GFP	72
	pDB59 P*agrP3-yfp*	*rnaIII* promoter controlling expression of YFP	73
	pJB128 (e.v.)	Insertless complement plasmid control (e.v.)	33
	pJB111 (AM)	Complement plasmid expressing the amidase domain of Atl (AM)	33
	pJB135 (GL)	Complement plasmid expressing the glucosaminidase domain of Atl (GL)	33
	pJB141 (Atl)	Complement plasmid expressing the full-length Atl (Atl)	33
Primers			
VJT1836	tetM.F	GATTGTAAAATAACAAATATTGGTACATG	This study
VJT1837	tetM.R	CAAAAGGTATCAATGAAGCAAGAAATATTG	This study
VJT3023	atl-up.F	CTCTAGAACTAGTGGATCCCCCGGGattagctataaagatgattcacaac	This study
VJT3024	atl-up.R	TACCAATATTTGTTATTTTACAATCtctatttattactcctaacatttattaattattac	This study
VJT3025	atl-down.F	TTTCTTGCTTCATTGATACCTTTTGgcaacatgaacataggatcaaaag	This study
VJT3026	atl-down.R	GCTGGGTACCGGGCCCCCCCTCGAGaataatctctctcttttaatgaagtc	This study

S. aureus cultures were grown in 5 mL of medium in 15-mL tubes with shaking at a 45° angle at 37°C. S. aureus was grown overnight in tryptic soy broth (TSB), and a 1:100 dilution of overnight cultures was subcultured into TSB. Unless otherwise specified, S. aureus was grown to early stationary phase (4.5 h of subculture) and was collected and normalized by optical density at 600 nm (OD_600_) for further experimental analysis.

### Generation of the *atl* mutant.

The Δ*atl* mutant was generated by replacing the *atl* locus with *tetM* gene encoding tetracycline resistance using the pIMAY* allelic exchange system (64). The primers are listed in Table 1. The Δ*atl* mutation was transduced into *ΔlukAB* background by phage 80α to generate Δ*lukAB* Δ*atl.* To complement the phenotype of *Δatl*, plasmids producing full-length Atl, the AM domain, or the GL domain (33) were electroporated into the Δ*atl* strain.

### Fractionation of bacterial culture.

The culture supernatant was prepared by trichloroacetic acid (TCA) precipitation. In brief, normalized bacterial cultures were pelleted by centrifugation at 4,000 rpm for 10 min. The culture supernatant was filtered through a 0.2-μm filter and precipitated at 4°C overnight with 10% (vol/vol) TCA. The precipitated proteins were washed with 100% ethanol, pelleted, air-dried, and solubilized with 8 M urea for 30 min at room temperature. The solution was mixed with 2× SDS sample buffer and boiled for 10 min.

To prepare the bacterial cell lysate, 1 mL of the normalized bacterial culture was washed with phosphate-buffered saline (PBS) and lysed with 100 μg/mL lysostaphin (Ambi Products LLC), 40 U/mL DNase, 40 μg/mL RNase A, and 1× Halt protease inhibitor (Thermo Fisher) in lysis buffer (10 mM MgCl_2_, 1 mM CaCl_2_ in 50 mM Tris, pH 7.5) for 30 min at 37°C. The lysate was mixed with 4× SDS sample buffer and boiled for 10 min.

To separate surface-exposed and membrane-proximal compartments, the OD_600_-normalized and washed bacterial pellet was incubated with 1× protease inhibitor in PBS for 10 min at 37°C, followed by incubation with 1% (wt/vol) SDS and 1× protease inhibitor in PBS for 30 min at room temperature. After centrifugation at 13,000 rpm for 2 min, the supernatant was collected as the surface-exposed fraction. The resulting pellet, namely, the membrane-proximal fraction, was washed three times with PBS containing 1× protease inhibitor and lysed as described above. Both fractions were mixed with 4× SDS sample buffer and boiled for 10 min.

### Coomassie staining and immunoblotting.

In 12% SDS-PAGE gels, equal volumes of protein samples prepared from OD_600_-normalized bacterial culture were loaded into each well, and proteins were separated by SDS-PAGE. To examine the exoprotein profile, the gels were stained with InstantBlue Coomassie protein stain (Expedeon). Quantification of the exoprotein profiles was performed using the Fiji distribution of the ImageJ software (65, 66). For each lane, a line with the same width of the lane was drawn vertically, and the profile of the line was recorded. The starting point of each line was normalized to the peak signal of the top band. The baseline was set to be the minimum value of each lane.

For immunoblotting, proteins were transferred onto a nitrocellulose membrane. The membrane was blocked with 5% milk, probed with rabbit anti-LukA (1:5,000) (16) or rabbit anti-LukE (for detecting the S subunit of other leukocidins) (1:5,000) (67) as the primary antibodies, and incubated with Alexa Fluor 680-conjugated goat anti-rabbit IgG (Invitrogen; 1:25,000) as a secondary antibody. Due to high sequence similarity, the anti-LukE antibody is able to detect the respective S subunits of HlgAB, HlgCB, LukED, and PVL. Images were acquired with the Odyssey CLx imaging system (Li-Cor Biosciences). Quantification of protein signals was performed using the Western analysis with the Image Studio software (Li-Cor Biosciences). Protein signals were normalized to WT or a purified recombinant protein control on each membrane.

### Infection assays.

Primary human neutrophils (polymorphonuclear neutrophils [PMNs]) were isolated from buffy coats from anonymous, consenting healthy donors (New York Blood Center) as described previously (68).

The PMN infection assays were carried out at the concentration of 2 × 10^5^ cells per well in RPMI without phenol red (Gibco) supplemented with 0.1% human serum albumin (HSA) and 10 mM HEPES in tissue culture-treated 96-well flat-bottom plates. The PMNs were infected with S. aureus at a multiplicity of infection (MOI) of 10 and incubated at 37°C in 5% CO_2_ for 2 h. Following infection, cells were pelleted by centrifugation at 1,500 rpm for 5 min, and lactate dehydrogenase (LDH) release was measured using the CytoTox-One homogeneous membrane integrity assay (Promega). In brief, 25 μL of culture supernatant was mixed with 25 μL of LDH reagent and incubated for 15 min at room temperature. Fluorescence was measured using a PerkinElmer 2103 EnVision multilabel plate reader (excitation, 555 nm; emission, 590 nm) and normalized to wells containing cells without S. aureus (0% cell lysis) and cells with 0.05% Triton X-100 (100% cell lysis).

### Cytotoxicity assays.

Bacteria were subcultured for 4 h, normalized to an OD_600_ of ∼1.2, and pelleted by centrifuging at 4,000 rpm for 10 min. The supernatant was filtered through a 0.22-μm filter. PMNs were seeded at 2 × 10^5^ cells per well in RPMI without phenol red (Gibco) supplemented with 10% fetal bovine serum (FBS) and incubated with different concentrations of culture filtrates at 37°C and 5% CO_2_. After 1 h, 10 μL of CellTiter 96 AQueous One solution (Promega) was added to each well, and the plate was incubated for another 1.5 h at 37°C and 5% CO_2_. The absorbance at 490 nm was measured using a PerkinElmer 2103 EnVision multilabel plate reader.

### Promoter reporter assays.

The promoter activities were measured using individual gene promoters fused to a superfolder green fluorescent protein (GFP) (*PlukAB*, *PlukSF-pv*, *Psae*) or yellow fluorescent protein (YFP) (*Pagr*). Bacteria were washed and diluted 1:2 with PBS. The GFP or YFP fluorescence and OD_600_ in 200 μL of the suspension were measured using a PerkinElmer EnVision 2103 multilabel reader. The GFP/YFP signal was normalized by the OD_600_ readings.

### Cytochrome *c* binding assay.

The positive surface charge was determined based on bacterial ability to repulse cationic protein cytochrome *c* as described previously (69, 70). Early-stationary-phase bacteria were adjusted to an OD_600_ of ∼1.2. Aliquots of 2 mL bacteria were washed twice with sodium acetate buffer (20 mM, pH 4.6). The pellet was resuspended in 0.5 mL 0.25 mg/mL cytochrome *c* in sodium acetate buffer and incubated with shaking for 15 min at 37°C. The bacteria were centrifuged at 13,000 rpm for 2 min, the supernatant was aliquoted, and the absorbance at 410 nm was measured using a PerkinElmer 2103 EnVision multilabel plate reader. The measurement was normalized to 0.25 mg/mL cytochrome *c* as the percentage of unbound cytochrome *c*, and then normalized to WT.

### Microscopy of cell clustering.

To visualize the cell shape of WT and *Δatl* by microscopy, washed bacterial cells were settled on a clean coverslip for 15 min and fixed with 4% paraformaldehyde (PFA) for 15 min at room temperature. The coverslip was washed three times and stained in the dark with 1 μg/mL BODIPY FL vancomycin (Invitrogen) in PBS for 10 min. The coverslip was washed three times, mounted with Fluoromount-G, and air-dried overnight in the dark.

Slides were imaged using Plan-Apochromat 63×/1.4 oil DIC M27 Elyra objective on a Zeiss 880 laser scanning confocal microscope with Airyscan. The BODIPY FL channel was imaged using 488-nm excitation and 495- to 550-nm emission filters. Z-stacks of fluorescent channels were collected at 0.17-μm steps to cover the depth of the bacterial cell clusters. Three-dimensional Airyscan processing was performed for raw images using the Zen software (Zeiss) with automatic strength. A single slice of brightfield was captured as a reference for bacteria position. Three random fields were collected for each condition in each experiment, and two independent experiments were performed.

Images were processed in the Fiji distribution of the ImageJ software (65, 66). Stack images were Z-projected by maximum intensity. The cluster areas were identified as fluorescent signal over 2,000 and including all holes. Each separate cluster was saved as a region of interest (ROI) and measured for area.

### Autolysis assay.

The autolysis assay was adapted from a previous study (50). In brief, overnight culture of WT and *Δatl* was washed and diluted 1:4 into PBS plus 0.05% Triton X-100 with or without 10 U/mL mutanolysin. The bacteria were grown at 37°C under shaking condition, and the OD_600_ was measured every 15 min using a Bioscreen C.
